# A bespoke rapid evidence review process engaging stakeholders for supporting evolving and time-sensitive policy and clinical decision-making: reflection and lessons learned from the Wales COVID-19 Evidence Centre 2021–2023

**DOI:** 10.1186/s12961-025-01297-w

**Published:** 2025-03-20

**Authors:** Ruth Lewis, Alison Cooper, David Jarrom, Mala Mann, Rebecca-Jane Law, Deborah Edwards, Judith Carrier, Hannah Shaw, Tom Winfield, Llinos Haf Spencer, Jane Noyes, Helen Morgan, Jennifer Washington, Elise Hasler, Micaela Gal, Elizabeth Doe, Natalie Joseph-Williams, Adrian Edwards

**Affiliations:** 1 Wales COVID-19 Evidence Centre, Health and Care Research Wales, Welsh Government, Cardiff, United Kingdom; 2 North Wales Centre for Primary Care Research, Bangor Institute for Health & Medical Research, North Wales Medical School, Bangor University, Bangor, United Kingdom; 3 Wales Centre for Primary and Emergency Care Research Wales, Division of Population Medicine, Cardiff University, Cardiff, United Kingdom; 4 Health Technology Wales (HTW), Cardiff, United Kingdom; 5 Specialist Unit for Review Evidence (SURE), Cardiff University, Cardiff, United Kingdom; 6 Technical Advisory Cell, Welsh Government, Cardiff, United Kingdom; 7 Ser Cymru, Welsh Government, Cardiff, United Kingdom; 8 Wales Centre for Evidence Based Care, School of Healthcare Sciences, Cardiff University, Cardiff, United Kingdom; 9 Public Health Wales, Cardiff, United Kingdom; 10 Centre for Health Economics & Medicines Evaluation, Bangor Institute for Health & Medical Research, School of Health Sciences, Bangor University, Bangor, United Kingdom; 11 Bangor Institute for Health & Medical Research, School of Health Sciences, Bangor University, Bangor, United Kingdom

**Keywords:** Evidence synthesis programme, Rapid reviews, Stakeholder involvement, COVID-19, Pandemic

## Abstract

**Background:**

The COVID-19 pandemic presented policymakers with time-sensitive decision problems and a rapidly increasing volume of research, not all of which was robust, or relevant to local contexts. A bespoke evidence review process supporting stakeholder engagement was developed as part of the Wales COVID-19 Evidence Centre (WCEC), which could flexibly react to the needs of decision-makers, to address urgent requests within days or months as required.

**Aims:**

To describe and appraise the WCEC review process and methods and identify key learning points.

**Methods:**

Three types of rapid review products were used, which could accommodate the breadth of decision problems and topics covered. Stakeholder (including public) engagement was integrated from the onset and supported throughout. The methods used were tailored depending on the needs of the decision-maker, type of research question, timeframe, and volume and type of evidence. We appraised the overall process and compared the methods used with the most recent and relevant best practice guidance.

**Results:**

The remote collaboration between research teams, establishing a clear pathway to impact upfront, and the strong stakeholder involvement embedded in the review process were considered particular strengths. Several key learning points were identified, which focused on: enhancing stakeholders’ abilities to identify focused policy-relevant research questions; the collection and storage of review protocols at a central location; tightening quality assurance process regarding study selection, data extraction and quality assessment; adequate reporting of methodological shortcuts and understanding by stakeholders; piloting of an algorithm for assigning study design descriptors, and a single quality assessment tool covering multiple study designs; and incorporate, where appropriate an assessment of the confidence in the overall body of evidence using GRADE or similar framework.

**Conclusions:**

The review process enabled a high volume of questions that were directly relevant to policy and clinical decision making to be addressed in a timely manner using a transparent and tailored approach.

**Supplementary Information:**

The online version contains supplementary material available at 10.1186/s12961-025-01297-w.

## Background

Health- and care-related policy and practice decisions should be based on relevant and trustworthy research evidence, but this relies on providing policymakers and their advisors with timely and accessible evidence [1]. Effective communication and collaboration between researchers, topic experts and decision-makers are key elements in achieving impact from research. The coronavirus disease 2019 (COVID-19) pandemic demanded new ways of working between academics, policymakers and others making health and social care practice decisions to address time-sensitive decision problems within an ever-changing environment and evidence base. Identifying and synthesising the rapidly increasing volume of available research evidence, not all of which was robust or relevant to specific local contexts, was an important challenge.

Systematic reviews represent the gold standard for informing policy and practice as they provide a comprehensive, rigorous and transparent synthesis of the evidence. They use standardised and empirically tested methods to minimise bias and error. However, they can take years to complete. One alternative approach is a rapid review – an abbreviated systematic review, where processes are streamlined or omitted, to produce evidence for policy and decision-makers in a timely (and resource-efficient) manner [2]. However, even rapid reviews can take 6 months or more to complete [3, 4], whilst policy and practice decisions were needed within days or weeks during the pandemic. Further rapid evidence review products, that either modify or use alternative methods, have been developed. Hartling et al. [5] developed a taxonomy of these products, based on the extent of synthesis conducted (Box 1), which includes four categories: evidence inventories, rapid response briefs, rapid reviews and automated products.

**Box 1:** Taxonomy of rapid review products

*Evidence inventories* – list of available evidence with no attempt to appraise, synthesise or present conclusions or recommendations (e.g. systematic maps, scoping reviews) *Rapid response briefs* – summary without formal synthesis of the best available evidence for addressing a specific question, generally based on the conclusions of existing synthesised evidence, such as systematic reviews and clinical guidelines *Rapid reviews* – appraisal and synthesis of the evidence for generating new conclusions using abbreviated systematic review methods for completion within a short time *Automated products* – computer programme generated analysis addressing user-defined questions derived from a database of evidence created using (unconnected) systematic search, screening and data extraction	

Hartling et al. [5] 10.1016/j.jclinepi.2015.05.036

Rapid evidence review products have demonstrated great utility for decision-makers, especially during the COVID-19 pandemic [6]. However, there are several key considerations in their development. Firstly, they are demand-driven and produced to support a specific decision by a particular end user [4, 5, 7]. This, and the timeframe of the decision problem, drives the choice of methods used [5]. Secondly, they require a continuous and close relationship with the end user, involving iterative feedback throughout the work [5], which is essential when restricting the scope of the review, to ensure the findings are directly relevant to decision-making [5, 7]. Thirdly, having a team that includes research staff experienced in systematic reviewing is critical for developing an expedited product [5]. Lastly, the COVID-19 pandemic, with its characteristic need for evidence to address rapidly evolving challenges, highlighted the need to avoid duplication across review groups.

The Wales COVID-19 Evidence Centre (WCEC) was established by the Welsh Government in March 2021 to enhance the use of research and evidence in managing the pandemic. It aimed to provide health and social care policy and practice decision-makers timely access to the latest relevant COVID-19 research evidence.

The purpose of this paper is to: (1) describe the bespoke evidence review process developed by the WCEC that takes account of the important considerations above, with the aim of supporting the agile and timely production of robust evidence reviews, whilst maintaining strong stakeholder engagement to ensure direct relevance to decision-making, and (2) appraise the overall review process and evidence review methods, their strengths and weaknesses, and identify further improvements that could be made.

## Methods

### The Wales COVID-19 Evidence Centre (WCEC)

The WCEC brought together a unique collaboration of established research groups within Wales with expertise in conducting rapid reviews, systematic reviews, health technology assessments, economic evaluations and the analysis of linked population-level routinely collected data. The WCEC operated through a core management team working closely (using videoconferencing) with the collaborating partner research teams (Box 2).

The WCEC undertook evidence reviews to address knowledge gaps and the specific needs of government, healthcare, public health and social care stakeholders in Wales. The evidence produced was designed to be of immediate use to decision-makers and to have a direct impact on decision-making, patient and client care, reducing inequalities and identifying future research needs. The work of the WCEC was delivered through four main processes: question prioritisation process, evidence review process, knowledge mobilisation process, and stakeholder engagement (including public involvement). This paper focuses on the evidence review process, and the stakeholder engagement that supports this. The processes for prioritising and setting research questions, and knowledge mobilisation, are described in more detail elsewhere [8, 9].

**Box 2:** Wales COVID-19 Evidence Centre (WCEC) collaborating partners

WCEC operated through a core management team working closely with six collaborating partners:	
• Health Technology Wales (HTW) – http://www.healthtechnology.wales/	
• Wales Centre for Evidence-Based Care (WCEBC) – A JBI Centre of Excellence – https://www.cardiff.ac.uk/research/explore/research-units/wales-centre-for-evidence-based-care	
• Specialist Unit for Review Evidence (SURE) centre – https://www.cardiff.ac.uk/specialist-unit-for-review-evidence	
• Public Health Wales Evidence Service – https://phw.nhs.wales/services-and-teams/observatory/	
• Bangor Institute for Health & Medical Research (BIHMR) – Centre for Health Economics and Medicines Evaluation – https://cheme.bangor.ac.uk/research/whess.php.en – in conjunction with Health and Care Economics Cymru (HCEC) – https://healthandcareeconomics.cymru/	
• Population Data Science – SAIL Databank – https://saildatabank.com/	
The core management team comprised a Director and leads for each of the four processes: prioritisation process, evidence review, knowledge mobilisation and impact, and stakeholder engagement. It worked closely (and remotely) with a public partnership group and members of the Welsh Government’s Technical Advisory Cell and Technical Advisory Group (TAC/TAG – sometimes referred to as “Welsh SAGE”) [10]. There was also a methodology subgroup, with representation from all collaborating partner groups, meeting on-line fortnightly for methodological support and to share good practice. Members of the public partnership group (PPG) provided public involvement in each review and are involved in the knowledge mobilisation process	

### Development of the WCEC evidence review process

The WCEC sought to develop an evidence review process that could deliver robust reviews within 4–8 weeks, but with flexibility to provide decision-makers with a credible summary of the available evidence within days or weeks when needed. We considered the range of rapid evidence review products identified by Hartling et al. [5] (Box 1), but we were also mindful to avoid having too many types of outputs, as this could be confusing to stakeholders [11]. We developed a phased reviewing approach [12, 13] which utilises three types of rapid review products: a rapid response product (which is called a rapid evidence summary), an evidence inventory product (called a rapid evidence map), and a rapid review. These are described in more detail in Table 1.

**Table 1:** Summary of the rapid review products included in the WCEC rapid evidence review process

	Phase I: rapid evidence summary (preliminary search of the literature; topic exploration)	Phase III: rapid review For broad policy questions, this may incorporate a two-staged process, where an initial descriptive map or scoping review is conducted to inform the focus of the rapid review	
Product type	Rapid response	Inventory	Rapid review
Timeframe	~1 week	~1–2 months*	
Output/format	*Key messages and annotated bibliography* (with links to full text)	*Rapid evidence map* Using abbreviated systematic mapping or scoping methods	*Rapid review* based on abbreviated SR methods
Purpose	Provide early access to evidence; gauge potential size and type of existing research; inform the rapid review methods/protocol/search strategy; support stakeholder involvement	Provide a description of the available evidence; identify substantial focus; identify existing research and evidence gaps	Provide a summary and direction of effect and possibly strength of the evidence
	*NB If an up-to date, robust and directly relevant evidence review is identified during phase I further review work may not be required; a critical appraisal and summary of the evidence review will likely suffice (with, if necessary, a limited update). Where multiple SRs are identified, these will be reviewed as part of the subsequent rapid review*		
Methods	A preliminary search of key resources (prioritising COVID-19 resources and sources of robust evidence syntheses)	Protocol and full search strategy developed Limitations on scope and comprehensiveness of review applied: limited number of sources searched; targeted grey literature; limited number of outcomes; study design restrictions (*Limits vary by topic, timeframe and extent of available evidence*)	
Data type	Based on abstracts of best available secondary/tertiary evidence	Based primarily on abstracts with some full text as required	Based on full texts
Integration of evidence	Reference list + key messages	Narrative summary of study characteristic	Narrative summary of study characterises and evidence synthesis
Risk of bias assessment	Not applicable	Not included	Yes (using validated instrument where feasible)
Limitations/disclaimers	• Not all relevant evidence will have been identified • Assessment based mainly on titles and abstracts • Quality of the listed/included evidence not assessed • Conclusions cannot be drawn	• Possible that not all relevant evidence identified • Quality of the listed/included evidence not assessed • No synthesis of results conducted; provide description of available evidence • Conclusions cannot be drawn	• Possible that not all relevant evidence identified • There may be potential biases in cutting corners (SR methods). (*Transparency in reporting and following a methodology will highlight limitations*)

*SRs* systematic reviews

^*^ The length of time may need to be extended in some instances and will depend on the breadth and complexity of the research topic/question(s), extent of the evidence base and type of analysis required to synthesise the evidence

### Best practice framework

Our overall process and methods development were informed by guidance for conducting and reporting rapid evidence review products [7, 11–18]. The methods selected for our rapid reviews were adapted according to the topic area, type of review question, the extent of the evidence base, urgency of the questions, and the needs of the decision-makers. To support the collaborating partner review teams, a best practice framework (Table 2) was developed with recommendations from key sources for methodological shortcuts that could be applied at each stage of the rapid review.

**Table 2:** Comparison of our rapid review methods with recommendations presented in the best practice framework

Best practice framework developed to inform rapid reviews conducted by the Wales COVID-19 Evidence Centre (WCEC)						Comparison of the methods used in WCEC reviews with the best practice framework recommendations
Review stages	Key published sources providing best practice guidance for conducting and reporting rapid reviews			Existing guidance developed by two WCEC collaborating partners for conducting rapid reviews		
	**Garritty, 2021 (Cochrane RR guidance)** [7]	**Tricco, 2017 (WHO) – key considerations** [13] (Production time usually ~1–4 months)	**Pluddemann, 2018 (Restricted SRs)** [18]** – minimum requirement [additional steps to reduce bias]**	**SURE—PACeRS (Mann, 2019)** [11] (Production time 8–10 weeks)*	**HTW** [19]** – evidence appraisal report (EAR)** (production time 3–6 months)**	**[OUTCOME CODING]** **Comments on the methods used in WCEC rapid reviews (RRs) and rapid evidence maps (REMs) **(production time usually ~2 months)
Question development & refinement	Involve key stakeholders to set and refine the review question, eligibility criteria and the outcomes of interest; to ensure the research question is fit for purpose; and regarding any ad hoc changes that may occur as the review progresses	Work with requester to ascertain intended purpose, scope and timeline Ensure the proposed approach fits the intended purpose	Clearly formulated research question, with rationale for why research is needed Patient, public and policy involvement, where resources permit	Rapid review request form template – incorporating PICO/SPICE framework; question refined with requestor and review advisory group (RAG)	Topic proposer involved in refining Q and identifying independent topic reviewers and other stakeholders. External topic advisor(s) (could be same as topic proposer) recruited to advice evidence review team	**[GREEN]** Key stakeholders and subject experts involved in: • Ascertaining intended purpose and timeline • Setting and refining research question, eligibility criteria • Feedback on review during/after the review process
Preliminary work to inform scope		Preliminary literature search can help inform conversations with requestor and scope		*Initial preliminary search for existing SRs conducted*	*Initial topic exploration report (TER) conducted, which gives an overview of the evidence that exists on a topic and used to decide if there is enough evidence to do an appraisal*	**[GREEN]** Rapid evidence summary conducted, which incorporates a preliminary literature search to ascertain if evidence is sufficient for RR; identify existing SRs; inform the scope/refinement of the research question; and decide on the methods for the RR (or if a REM is needed)
Protocol development and approval	Develop a protocol that includes review questions using PICOS, or other framework, and details inclusion and exclusion criteria Protocol should be published (e.g. on PROSPERO)	*RR producers typically use a PICO format and develop key questions iteratively with requesters* Register protocol with PROSPERO and include “rapid review” or similar term in the title Use PRISMA reporting items	Basic protocol published on register, e.g. PROSPERO (reviewed by research team, potentially peer review publication)	Registered on PROSPERO where appropriate	Use of PROTOCOL template, incorporating PICO framework Published on-line; not registered	**[GREEN and RED]** Protocol includes review question(s) using PICOS, or other framework, and details inclusion and exclusion criteria. Protocol is developed with input from stakeholders and made available on request (which is noted in the final report). Protocol is not published on register
Conceptual framework		Use a conceptual framework for complex Qs relating to health policy and system improvements		For complex interventions, logic models may be used to help define Q and inform review		**[AMBER]** Conceptual framework used in a couple of reviews; not required in most reviews
Search	Involve an information specialist Main databases to search: MEDLINE, CENTRAL and Embase; plus (if required) two additional specialist database/sources Limited grey literature and supplemental searching	Tailor database selection to topic (commonly used: PubMed/MEDLINE, Cochrane Library and Embase). Add grey literature search depending on topic, purpose and timeline Use staged search to first identify existing SRs, then studies with other designs that provide the most rigorous evidence to answer questions Peer review search strategy, using a tool e.g. the PRESS checklist	At least one major scientific database and one other source (e.g. topic specific database or Google Scholar) Limit by date and language (acceptable) Use previous review as starting point (acceptable) (No date limit Include unpublished studies, grey literature No language limit)	Searches conducted by experienced information specialists *Initial search for existing SRs: CDSR, PubMed – Clinical Queries* + *Health* Main search: 3–4 databases (MEDLINE, CINAHL, Cochrane Library, Embase, HMIC, JBI EBP and PsycINFO), eTOC's of key journals, citation tracking and grey literature if time available Initially search limited to last 5–10 years depending on literature, English language and studies published in Organisation for Economic Co-operation and Development countries; no study design restriction. Excluding conference abstracts, doctoral dissertations and book chapters Search strategy developed on MEDLINE and first 20 hits sent to requestor for checking relevance	Searches conducted by experienced information specialists Selection of databases and other sources informed by literature search SOPs; database checklist Unpublished literature not considered	**[GREEN]** Searches conducted (or informed) by experienced information specialists and reviewed by the wider team (no formal peer review e.g. using PRESS checklist was conducted). Minimum set of databases, tailored to question/topic area, limited grey literature searched. (Some reviews incorporate more extensive grey literature searches, depending on topic, purpose and timeline, and informed by the stakeholders)
Study selection	Screening title and abstract – two reviewers to dual screen at least 20% of citations, resolving all conflicts. One reviewer to screen remaining citations and one to review all excluded citations, resolving all conflicts if needed Full text – one reviewer to screen all manuscripts and one to review all excluded manuscripts Use standardised forms; pilot for calibration and test use across the whole team. Consider using SR software	In lieu of dual screening and selection, use a single experienced reviewer for application of inclusion criteria and two reviewers for application of exclusion criteria, or one person for screening ,with verification of a subset of records by another	Single reviewer of titles, abstracts and full texts (sufficient) Verification of a random sample of full texts by a single reviewer (acceptable) (Verification of a random sample of full texts by a second reviewer All full texts by two reviewers Titles, abstracts and all full texts by two reviewers]	Screening title and abstract – two independent reviewers (*Mann, 2019*) Full text – study selection carried out by one reviewer and checked for accuracy by another		**[AMBER]** Single reviewer screening in most cases – sometimes with “add ons” e.g. internal quality assurance, verification of a sample or excluded citations/manuscripts. Some reviews include dual (independent) citation and full text screening,
Data extraction	Single reviewer to extract data (using piloted form), with second reviewer checking for correctness and completeness. Consider splitting data extraction into two parts: (i) study characteristics, extracted by a single reviewer; and (ii) outcome data extracted by two independent reviewers Limit extraction to a minimal list of required items Consider using data from existing reviews to expedite data extraction	Use a single reviewer to extract data, with a second reviewer checking ≥ 10% random sample for accuracy. Dual performance or checking may be needed more for quantitative results than descriptive study information Limit extraction to key study characteristics and outcomes	Single reviewer (sufficient) Verification of data extraction of a random sample by a single reviewer (acceptable) (Verification of data extraction of a random sample by a second reviewer All data extraction verified by a second reviewer Seeking additional data sources)	Data extraction carried out by one reviewer and checked for accuracy by another *(data extraction forms based on PICO framework)*		**[AMBER]** Single reviewer data extraction with quality assurance/verification of a sample. All data extraction checked in some
Critical assessment of included studies/risk-of-bias	Use a valid risk of bias (RoB) tool specific to the study design(s) Single reviewer to rate RoB, with full verification of all judgments by a second reviewer Limit RoB ratings to the most important outcomes	Choice of appraisal instrument varies in practice, with both standard and customized approaches used An approach similar to that for data extraction can be used (i.e. single reviewer, with verification by a second reviewer)	Single reviewer (sufficient) Rapid appraisal tools (acceptable) (Partial verification of the risk of bias assessment by a second reviewer. All risk of bias assessment verified by a second reviewer Detailed appraisal tools)	Quality assessment using amended version of GATE checklist (*Mann, 2019*) incorporating both internal and external validity; different checklists for quantitative and qualitative studies (or range of SURE critical appraisal checklists for different study design) Each study assessed by one reviewer and checked for accuracy by another	No formal quality appraisal process, but the EARs include an informal assessment of evidence quality, taking into account generalisability, applicability, sources of bias and any other relevant strengths or limitations. Existing checklists may be used to prompt items for consideration and inform author’s conclusion	**[GREEN and AMBER]** Quality assessment tools specific to the study designs used in most cases and results synthesised in a narrative; individual study appraisal findings documented as an appendix or available on request Critical appraisal generally conducted by a single reviewer, with findings often verified by a second for a sample or all
Synthesis	Synthesize evidence narratively. Consider meta-analysis only if appropriate (i.e. studies are similar enough to pool) using appropriate standards	An iterative approach to the synthesis process can involve post hoc protocol adjustments	Narrative synthesis (sufficient) Summary tables (required) (meta-analysis of all outcomes)			**[GREEN]** Narrative synthesis only. This was sometimes limited to a descriptive summary of studies and their results, rather than a full synthesis. Summary tables provided
Assessment of body of evidence	Single reviewer to grade the certainty of evidence, with verification of all by a second reviewer	The quality of the body of evidence and the strength of any recommendations can be assessed using an approach such as the GRADE system Limitations of the review should be discussed and cautious conclusions provided		Reported key findings includes documenting: reliability, consistency and relevance of evidence		**[AMBER]** Variable use of GRADE to assess certainty of the evidence; overall body of evidence often assessed in a narrative without using a tool
Report productions		Software tools can help automate and track review steps Standardisation of processes and templates aids in production of the report and enhances transparency of the review Final reports often include implications, recommendations for policy and discussion of research limitations	Basic publication without appendices and added data (sufficient) (Publication without appendices and added data)	Report format can vary greatly from generation of a reference list through to detailed appraisal Format and template informed by initial workshop and approved by end-users, incorporating: (1) Methods + context; (2) key findings split into: reliability, consistency, and relevance of evidence; (3) policy and clinical implications; (4) PRISMA flow diagram; (5) table of study summaries; and (6) list of included studies Draft report reviewed by RAG; revised and sent to requestor for response; queries addressed liaising with RAG; second draft submitted to requestor; final review document developed liaising with requestor	Reporting template used EARs do not make recommendations for NHS Wales, but HTW’s Appraisal Panel may issue guidance based on the report’s findings External peer review conducted	**[GREEN]** Reporting template used. Reports include implications, recommendations for policy and research limitations. Appendices used where needed
Dissemination/knowledge translation		Peer review journal publication infrequent		Reviews added to the PaCERS RR repository (available online), and distributed to decision-makers via e-mail	All topic exploration reports and evidence appraisal reports available online Sometimes published as peer review journal publications	**[GREEN]** Published online and preprint servers (e.g. medrxiv), and disseminated via comms/stakeholders/briefings/symposiums

As part of the methods appraisal, recommendations in the best practice framework were colour coded green: where WCEC methods met or exceed the recommendation; orange: where some WCEC methods met or partially met the recommendation; or red: were the majority of WCEC methods did not meet the recommendation. The outcome of this appraisal is summarised here as text in the last column (N.B. Multiple recommendations were made by each guidance for individual review stages included in the framework.)

*CDSR* Cochrane Database of Systematic Reviews; *EAR* evidence appraisal report; *GATE* Graphic Appraisal Tool for Epidemiological studies; *GRADE* Grading of Recommendations, Assessment, Development and Evaluations; *HTW* Health Technology Wales; *NHS* National Health Service; *PaCERS* palliative care evidence review service; *PICO* participants intervention comparator outcomes; *PICOS* participants intervention comparator outcomes study design; *PRISMA* Preferred Reporting Items for Systematic Reviews and Meta-Analyses; *Q* question; *RAG* review advisory group; *RoB* risk of bias; *REM* rapid evidence map; *RR* rapid review; *SOPs* standard operating procedures; *SPICE* setting population or perspective intervention comparison evaluation; *SR* systematic reviews; *SURE* specialist unit for review evidence; *TER* topic exploration report; *WCEC* Wales COVID-19 Evidence Centre; *WHO* World Health Organization

^*^ palliative care evidence review service (PaCERS) use rapid review, which are defined as “a review conducted within 8–10 weeks using modified systematic review methods with a highly refined research question, search carried out within limited set of databases and other sources and increasing the transparency of the methods used”

^**^ Health Technology Wales (HTW) evidence appraisal reports (EARs) are not comprehensive systematic reviews or full technology assessments. They are based on the best evidence that Health Technology Wales can identify and retrieve within the time available using rapid review methodology

Three key guidance documents were prioritised for developing the framework summarising the recommendations for best practice of conducting a rapid review [7, 13, 18]. We also referred to two existing guidance documents, developed and already used by two collaborating partners for conducting rapid reviews [11] or rapid health technology assessments [19].

### The review process

**Fig. 1:**
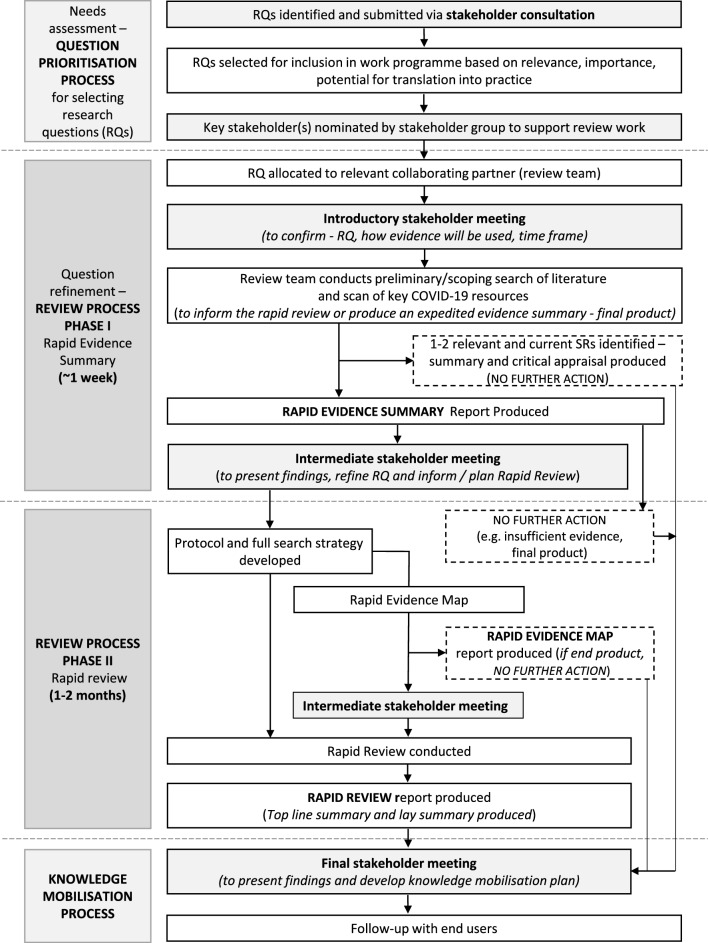
WCEC rapid evidence review process

The phased review process is outlined in Fig. 1 and described in more detail in the next section. Each review was conducted by a dedicated collaborating partner review team supported by the core management team. A continuous and close relationship with the decision-makers and relevant stakeholders (including public partnership group representation) was facilitated by three or more online stakeholder meetings.

### Question prioritisation process

The review question(s) were submitted by stakeholders (e.g. policymakers/advisors, health and social care leads, public, academic/research groups) and prioritised during a formal consultation process, which is reported in detail elsewhere [9]. Urgent questions could also be submitted directly by policymakers or TAC/TAG members and fast-tracked onto the WCEC work programme. Key stakeholders, including those submitting the question and members of the public partnership group (PPG), provided expert (topic and methodological) input throughout the evidence review process. The overall review process and commitment required (including attendance at online meetings) was explained to the stakeholders submitting the question at the onset, and it was made clear that we were unable to take on questions where this stakeholder commitment was not feasible.

### Review process phase I: rapid evidence summary (RES)

In phase I, the review question was allocated to an appropriate WCEC collaborating partner (review) team, and an introductory stakeholder meeting organised. This early phase comprised preliminary work to inform the rapid review work. However, it was adaptable to produce a final rapid response product (Table 1) within weeks if no rapid review was planned.

#### Introductory stakeholder meeting

The stakeholder meetings included members of the core management team and WCEC public partners, the review team and relevant stakeholders. The *introductory* meeting was used to confirm the decision problem or review question including key outcomes, clarify how the evidence would be used and confirm required timelines. It was also an opportunity for stakeholders to notify the review team of potentially seminal research or useful grey literature sources. Where an ill-defined decision problem/question had been submitted in the prioritisation process, this meeting also served to develop a structured review question.

#### Preliminary search of the literature

The review team then conducted a scoping search and a scan of key COVID-19 resources. This was supported by a tailor-made resources list, including both COVID-19 specific and generic registries and databases of secondary research (Supplementary Information, Additional file 1). This preliminary review of the literature enabled the reviewers to familiarise themselves with the topic area, check the research question has not been addressed by other groups or evidence centres, identify the extent and type of available evidence, and inform the methods and design of the rapid review in phase II (and develop the protocol). The searches focused on identifying robust secondary or tertiary research. Primary studies were considered if no relevant reviews were identified. The extent of the search was adapted according to whether this stage represented the final output or not.

#### Output from phase I

The output from this first phase was presented as an *annotated bibliography* with key findings, using a template to support the efficient and transparent reporting of what was done and found. When there was a high priority urgent decision to address, or insufficient evidence for a rapid review, the rapid evidence summary was published as the final output for the stakeholder. For example, our review of ozone machines and other disinfectant in schools (RES_23) [20].

If an up-to date, robust and directly relevant evidence review or clinical guideline was identified during the preliminary searches then a critical appraisal and summary of the review was conducted. For example, our review of vaccination in pregnant women (RES_24) [20]. If multiple systematic reviews were identified, then a review of existing reviews was considered for the subsequent phase rapid review. For example, in our review of innovations to support patients on elective surgical waiting lists (RR_30) [21] and our review of interventions to recruitment and retain clinical staff (RR_28) [22].

#### Intermediate stakeholder meeting

The findings of the initial phase (if progressing to a rapid review) were presented at a second, *intermediate*, stakeholder meeting. Collaborative discussions refined the review question, drafted eligibility criteria and decided on the overall reviewing approach to be used (if proceeding to rapid review). Stakeholders identified important contextual issues, known equality, or economic impacts for consideration in the proposed review.

### Review process phase II: rapid review

Phase II comprised a rapid review (RR) of the evidence, usually completed within 1–2 months. This could be supplemented or substituted by a rapid evidence map (REM). The rapid review delivered a synthesis or meta-synthesis of the evidence, whilst the rapid evidence map provided a description of the available literature (Table 1). Both were based on a comprehensive search strategy and pre-defined protocol.

#### Rapid evidence map

For broad or complex review questions a rapid evidence map could be conducted, providing an inventory of the nature, characteristics and volume of available evidence for the particular policy domain or research question. The rapid evidence map was based on abbreviated systematic mapping [23] or scoping review [24] methodology, depending on the type of review question. For example, our review of recruitment and retention of NHS workers [20]. Stakeholders could also request a rapid evidence map as the intended final rapid product. For example, in our review of inequity experienced by the LGBTQ+ community [20].

#### Rapid review

Our rapid reviews used an adapted systematic review approach, with some review components abbreviated or omitted to generate the evidence to inform stakeholders within a short time frame, whilst maintaining attention to bias. We followed methodological recommendations and minimum standards for conducting rapid reviews [7, 13, 18]. The approach and decisions made on tailoring the rapid reviews were the responsibility of the individual review teams, according to the type of question, research volume and time frame, in discussion with core management team members and expert stakeholders.

#### Output from phase II

The template for our final rapid review and rapid evidence map reports are based on recommendations for reporting evidence reviews for decision-makers [11, 16]. This incorporates a two-page “top line summary”, the results and recommendations for practice presented up front, and the details of the methods used at the end of the report. The report also included a section of “additional information” where the input from the stakeholders was acknowledged and any conflicts of interest that the authors had was noted.

Our review reports were made available via a library on the WCEC website [20]. From May 2022, reports were published on a pre-print server and allocated a doi. Thus, reports could be identified readily in database searches, and other review teams could identify potential duplicate review questions early on. A short lay summary and the links to the pre-print server were included in the WCEC library. The ongoing WCEC work programmes, which included questions in progress, scheduled and completed, was also published on the website.

### Knowledge mobilisation process – planning pathway to impact

#### Final stakeholder meeting

A *final* stakeholder meeting was used to present the findings of the review to the stakeholders, address any queries, identify the policy and practice implications, and support the development of a knowledge mobilisation plan.

### Appraisal of the overall review process and rapid review methods

We appraised our overall approach and rapid review methods to reflect on our experience of implementing the WCEC review process and to identify key learning points.

We compared our methods and practice with the recommendations of Garritty et al. [7], Tricco et al. [13], Plüddemann et al. [18], Mann et al. [11], and Health Technology Wales [19], as the principal resources for our own best practice framework (Table 2). We also compared our rapid review methods with the array of methodological shortcuts recommended in published guidance developed or used across rapid review centres and organisations, as reviewed by Speckemeier et al. [25] (Table 3). That scoping review included guidance for any type of rapid evidence product with a completion time ranging from a day to over 6 months. The output included a table summarising the range of recommendations, or methodological shortcuts, provided in the guidance, and the frequency with which they were reported. However, the authors did not provide an indication of which recommendations were optimal.

**Table 3:** Comparison of rapid review methods with range of recommended shortcuts in scoping review of published guidance

Development step*	Range of recommendations for methodological shortcuts in published guidance (most common in bold type, but not necessarily the optimal approach)	No. of guidance (*n* = 38)**	Approach (and range of methodological shortcuts) used in WCEC RRs***
Search strategy	• **Specific search strategy to address clearly defined question**	**21**	**Specific search strategy developed to address a clearly defined question** (with pre-defined eligibility criteria specified in a protocol)
	• Sensitive search strategy	5	
	• Individual adapted search strategy	3	
Databases searched	**• 3 or more databases**	**12**	Searches included > **3 databases**
	• 1–2 databases	7	
	• Search in limited number of databases	5	
	• Preliminary search to identify databases	5	
	• Individual choice of databases	2	
Search timeframe	• Search timeframe limited to 10 years	3	Search timeframe **customised according to research question** and selected in consultation with stakeholders. (The process is supported by the findings of the preliminary RES***)
	• Search timeframe limited to 5 years	4	
	• Search timeframe limited to 3 years	1	
	• **Customise search timeframe according to research question**	**10**	
	• No restriction	3	
Languages	• Limited to English and one further language	2	**Most limited to English language**, but some also included one or more further languages
	• **Limited to articles in English language**	**20**	
	• Individual restriction	5	
Search of grey literature	• **Inclusion of grey literature**	**12**	Customised approach used, **with grey literature search added** depending on topic, purpose and timeline (decision informed by stakeholders and preliminary RES***); can be extensive in some reviews
	• No grey literature search	8	
	• Customised approach	7	
Study design	• Search without restriction of study design	1	**Priority given to locating and summarising evidence from relevant and/or high-quality SRs** (or RRs, clinical guidelines, meta-analyses, HTA reports). However, on the basis of the findings of the preliminary RES*** the subsequent RR could focus on SRs only, SRs plus recent primary studies, or exclusively on primary research (with either unrestricted or individual choice of study design)
	• **Prioritising search for systematic reviews, meta-analyses, HTA reports and guidelines** (*expanding to primary studies if non identified*)	**25**	
	• Exclusive search for systematic reviews (SRs) and RCTs published after the most recent systematic review	6	
	• Individual choice of study design to include	1	
Inclusion of easily obtainable literature	• **Only easily obtainable literature is included**	**12**	**All available literature included** in most reviews, with existing reviews used where feasible
	• All available literature is included	4	
	• Customised approach depending on availability of evidence	2	
Full-text analysis	• **Full-text analysis**	**30**	All RRs based on **full-text analysis**. The REMs generally based on abstracts (with some full text assessment), depending on the requirements of the stakeholders, time frame and volume of research. (Preliminary RES* is based on titles and abstracts)
	• Analysis on abstract level	2	
	• Customised approach	2	
Screening: number of reviewers	• Screening carried out by two persons and uncertainties clarified through discussion	8	Range of shortcuts used for study selection, depending on the review question, the volume and type of evidence, and proposed timeframe: • Screening (citations and full text) carried out by one person • Screening carried out by one person with sample screened by a second • **Screening carried out by one person with uncertainties or sample of full text decisions checked** • Screening carried out by two (or more) persons with disagreements discussed • Citation screening carried out by one person and full text by two persons with disagreements resolved by third • Citation screening by one person with 20% reviewed by two people; full text screened by one person with excludes checked by a second • Citation screening by one person with 20% reviewed by two people and ≥ 50% of excludes checked by second person; full text screened by two independent people
	• Screening is carried out by one person and 20–25% of articles reviewed by a second person. If the match is < 95%, all articles screened independently by a second person	5	
	• Screening carried out by one person and second person screens excluded articles (in some cases only for title and abstract screening, while the full text is screened by two people)	3	
	• **Screening carried out by one person and a second person consulted in case of uncertainties**	**13**	
	• Customised approach	1	
Extraction: number of reviewers	• Extraction carried out by one person and all results verified by a second person	6	Range of shortcuts used for data extraction depending on the review question, the volume and type of evidence, and proposed timeframe: • **Extraction carried out by one person** • Extraction carried out by one person with sample checked by a second • Extraction carried out by one person with 10% double extracted for discrepancies • Extraction carried out by one person with all checked by a second • Extraction carried out by two independent persons
	• Extraction carried out by one person and results partially verified by a second person	7	
	• **Extraction carried out by one person**	**14**	
	• Customized approach	2	
Risk of bias assessment	• **Risk of bias assessment**	**20**	**Risk of bias assessment** conducted with validated or recognised risk of bias or critical appraisal tools in most reviews. No quality assessment conducted in a few RRs, but narrative summary of key limitations provided
	• No risk of bias assessment	7	
	• No independent assessment of the risk of bias but bias potential reported in the included evidence is incorporated	5	
	• Customized approach depending on availability of evidence	3	

*HTA* health technology appraisal, *RCT* randomised controlled trial, *RES* rapid evidence summary, *RR* rapid review, *SR* systematic review

Reference (scoping review): Speckemeier et al. [25]

^*^ This table is based on “Table 1: Recommendations for methodological short cuts” reported in the manuscript by Speckemeier et al. [25]. The following steps for developing a review, which are incorporated in our best practice framework and appraisal of our methods, were not included in the table by Speckemeier et al. [25]: risk of bias assessment – number of reviewers, synthesis and assessment of body of evidence

^**^ Not all guidance documents reported on each development step

^***^ The methods used within each of our rapid reviews and rapid evidence maps are presented in the Supplementary Information, Additional file 2. The methods used within individual reviews will vary, depending on the review question, the volume and type of evidence, and proposed timeframe. The approaches used are informed by a prior scoping review of the literature (presented as a rapid evidence summary, RES) and developed in collaboration with the stakeholders.

### The approach used for appraising our rapid review methods

We assessed whether our reviews, mainly completed within 2 months, aligned with our best practice framework, and whether methods aligned across our different collaborating partner groups. Findings were presented at a methods subgroup meeting and discussed to reflect on what worked well or could be improved (and how).

As part of this appraisal, key data from all rapid reviews and rapid evidence maps completed up until March 2023 were extracted. These included data on the search date, overall reviewing approach, limits applied, sources searched, volume of research identified, study selection process, data extraction process and approach used for quality assessment. An important consideration here is that the approach used depended on the research question being addressed, the volume and type of research available, and the timeframe within which the review was conducted.

Where the methods of individual reviews met or exceeded the recommendation in the best practice framework the text was highlighted green, for recommendations that were either partially or not always met the text was highlighted amber, and where our methods consistently did not meet the recommendation, the text was highlighted in red. We did not seek to identify individual failures or the frequency with which our methods did not meet the recommendations, but to reflect on our overall process and methodological approach used and identify what changes could be made. The colour coded Framework table was presented at a methods group meeting, and participants given a copy of the data extraction table summarising individual reviews.

## Results

### Results of the appraisal of our methods

The comparison of the methods used in our reviews with the recommendations in the best practice framework is presented in Table 2 as an additional column to the best practice framework. The full details of the methods used within our rapid reviews and rapid evidence maps are available in the Supplementary Information, Additional file 2. The comparison of our methods with the range of recommendations identified in the scoping review of guidance conducted by Speckemeier et al. [25] is presented in Table 3.

We identified that our basic methods align with or exceed most recommendations for rapid reviews, notably for developing and refining the review question, the use of preliminary work to inform the scope, the searches, synthesis and report production (Table 2). A potential gap was that, although our reviews are based on pre-defined protocols, which are developed in collaboration with the stakeholders, these are not registered. However, our protocols are made available on request, which is noted in the reports.

Study selection and data extraction were conducted by two independent reviewers in some reviews, but were more usually conducted by a single reviewer with or without verification of a sample or excluded citations/manuscripts. Quality assessment was based on critical appraisal or risk of bias tools specific to the study design(s), which agreed with most recommendations, but the assessment was often conducted by a single reviewer with or without a verification of a sample. The selection of literature, data extraction and critical appraisal by a single reviewer meets the minimum requirements only [18], and verification sample or the use of two independent reviewers is generally recommended to reduce bias [7, 13, 18]. The assessment of the confidence in the evidence base was generally subjective. The limited number of studies and diversity of outcomes reported in some reviews meant that the GRADE (Recommendations Assessment, Development and Evaluation) [26] assessment was applied to single studies. This was also the reason why some reviews did not include a GRADE assessment.

An important limitation identified in a minority of our earlier reviews is that the methodological shortcuts were not stated or clearly described. This is an important consideration for transparency and validity.

### Reflection on our methods and reviewing approach and identification of key learning points

The output of the methods appraisal was shared with the review teams at a methods subgroup meeting. Members were also asked to reflect on their experience of the overall review process.

Aspects of the overall process that were thought to be working well included the stakeholder process for formulating relevant questions and the facilitation of the stakeholder meetings. The methodological discussions that ensued between the WCEC core team and the review team, on planning and conducting the proposed reviews, were also valued. These were felt to be beneficial for problem solving and learning from each other. The remote working and cross Wales collaboration were also considered a strength, as were the published reports and impact strategy. Establishing a clear pathway to impact was also key for refining the review question. Both these stages could be supported by a network of policy decision-makers with enhanced abilities in both question formulation and impact work.

Each review was completed by a dedicated collaborating partner team with a resource allocation equivalent to two full-time researchers plus some senior input time. Each collaborating partner had a slightly different set-up, and the resource allocation was subdivided among multiple reviewers in some teams. However, there was limited capacity to append additional personpower where the review needed to be completed over a shorter interval, or when the extent of the literature was larger than anticipated. Rather the overall process was designed to support restricting the scope of the review in close collaboration with the stakeholders, developing of an initial evidence map and tailoring the review methods. The duration of the review could, however, be extended by about a month where the stakeholder timeframe allowed this. The collaborating partners included established research groups with expertise in systematic reviews, scoping or mapping reviews, rapid reviews and economic evaluation. The researchers conducting or leading the reviews were experienced reviewers, but inexperienced researchers were also given the opportunity to get involved and develop new skills. The review teams were also supported by a structured overall process, the use of reporting templates and regular methods group meetings.

The administration of support, and people’s enthusiasm and commitment to the overall process, was paramount. For example, the timing between the preliminary and intermediate meeting was tight and was achieved utilising various approaches depending on the review team and stakeholder requirements. This included, for example, checking at the start with stakeholders that they could still commit to the overall process; setting up a doodle poll that covered sufficient dates to allow both meetings to be set up from the onset; asking for people’s availability for organising the second meeting as part of the first on-line meeting; or circulating a separate short doodle poll for individual meetings on the basis of the availability of key people. The optimum approach was generally selected after the initial conversations with the stakeholder(s), and the review team confirmed. However, the timing had to be extended in some reviews to account for additional requirements of the preliminary review or people’s limited availability (e.g. due to sickness).

In terms of our methods, members acknowledged potential discrepancies between reviewers in allocating study descriptors, in particular for poorly reported or less robust study designs. The algorithm developed by Leatherdale [27] for assessing natural experiments and to inform selection criteria was noted as a potential solution, requiring evaluation. The use of a single checklist for assessing the risk-of-bias covering multiple study designs (addressing the same type of question) was considered potentially beneficial. However, using the validated checklist developed for any non-randomised comparative study of interventions, ROBINs-I [28], was considered challenging within the context of a rapid review and mainly applicable to identifying bias in studies assessing causal effects of interventions. Likewise, GRADE works best for assessing the confidence in the overall body of evidence for interventions that have been evaluated by randomised trials and where there is at least one meta-analysis to provide a single estimate of the outcome effect [7]. Our reviews cover various forms of evidence, including intervention effects, prevalence, prognostic, diagnostic, economic, meaningfulness and consequence of public health measures. The use of GRADE in very rapid reviews, in particular non-intervention reviews, was considered challenging, even though it is recommended for use in emergency settings, such as the COVID-19 pandemic [29]. Members acknowledged that it should be included where possible. It was acknowledged that adhering to the minimum standards, such as regarding single reviewer screening of the literature or data extraction, could lead to bias or inaccuracies. The need to adequately report the methodological shortcuts used and the limitations of the review was also re-iterated. The potential value of more in-depth reviews, closer to systematic reviews in methodology (and including for example, network meta-analysis, meta-ethnography or economic modelling), and taking longer to complete when required, was identified. The learning points are summarised in Box 3.

**Box 3:** Key learning points for rapid evidence synthesis with impact

• There is a need for a network of key stakeholders with enhanced abilities to identify focused policy-relevant research questions. The provision of training in developing focused research questions may also be beneficial • Identifying how the evidence is going to be used during the introductory stakeholder meeting and establishing a clear pathway to impact was key for refining the review question (or narrowing the scope of the review) • The continuous stakeholder involvement embedded within our review process was a particular strength, facilitated by remote working and close collaboration between different research groups and organisations across Wales • The core management team should collect protocols for all reviews to support making them available on request • Agreed in-house minimum standards are needed for the quality assurance processes, whilst acknowledging that these may be adapted according to the review question type, evidence base available, stakeholder needs and time available. Our reviews should align, where possible, with the minimum standards recommended in the Cochrane guidance for rapid reviews of interventions (Garritty et al. [7]; Garritty et al. [30]), and include: • *Screening title and abstract* – two reviewers to dual screen at least 20% of citations, resolving all conflicts. One reviewer to screen remaining citations and one to review all excluded citations, resolving all conflicts if needed • *Screening full text* – one reviewer to screen all manuscripts and one to review all excluded manuscripts • *Data extraction* – single reviewer to extract data (using piloted form), with second reviewer checking for correctness and completeness • *Risk of bias assessment* – single reviewer to rate risk of bias, with full verification of all judgments by a second reviewer • It is important to adequately report the methodological shortcuts used in our reviews and the limitations of the review. An understanding of these by the stakeholders is also essential to establish trust in the reviews • The algorithm developed by Leatherdale [27], for assessing natural experiments, may be useful to assign study design descriptors and inform the selection of study types for inclusion • A single quality appraisal tool that covers multiple study designs may be useful for reviews of intervention effects • The GRADE system for assessing the confidence in the overall body of evidence for each outcome should be used, where possible	

## Discussion

### Summary of the practice and its appraisal

The Wales COVID-19 Evidence Centre developed a review process that could flexibly react to the needs of decision-makers, to address urgent requests within days, weeks or months as required. For each review, the approach used, and methodological shortcuts applied, were tailored depending on the needs of the decision-maker, timeframe, and volume and type of evidence. A best practice framework, which integrates recommendations in key published guidance, was developed to support reviewers at each stage of the reviews.

We appraised our overall process and methods used in 27 rapid reviews and five rapid evidence maps. Our methods aligned with or exceeded most recommendations for conducting rapid reviews, particularly those for developing and refining the review question, undertaking preliminary work to inform the scope, conducting the searches, quality assessment, narrative synthesis and report production. However, our review protocols were not registered, and study selection, data extraction and quality appraisal were generally conducted by a single reviewer, and the assessment of confidence in the evidence base was generally subjective.

### The wider context of the literature

Several publications describe the rapid evidence review methods and overall process used in other centres [16, 31, 32]. The guidance and methods developed by these publications were also considered as part of a recent scoping review by Speckemeier et al. [25]. Our methods align with or exceeded the recommendations for methodological shortcuts most frequently reported in published guidance.

The trade-off in achieving speed and efficiency in conducting a rapid review is a reduction in the validity of the results and certainty in the evidence [25, 33]. However, empirical evidence of the impact of using specific methodological shortcuts is limited, and few shortcuts are used consistently in rapid reviews [4, 25, 33–35]. There is little consensus over which shortcuts could apply across different topic areas [4, 25, 33–35]. There is evidence showing that limiting the search strategy can increase the risk of selection, retrieval and publication bias [25]. The selection of literature and data extraction by a single reviewer can lead to relevant studies being missed and inaccuracies in data extraction [25, 33]. However, the extent of this impact varies depending on reviewer experience and research topic [25, 33, 36–38]. A crowd-based randomised trial [39] found that single-reviewer abstract screening missed on average 13% of relevant studies, and dual-reviewer screening missed 3% of relevant studies. It is important that the type and extent of the methodological shortcuts used are clearly reported, so that the extent of the potential bias and limitations of a review can be assessed.

The Cochrane Rapid Reviews Methods Group advocates that the essential element to success is early and ongoing engagement with the research requester to focus the rapid review and ensure that it is appropriate to the needs of stakeholders [7, 30, 33]. The stakeholder involvement process in our reviews was considered an important strength, facilitated by remote working and close collaboration between different research groups and organisations across Wales. A potential limitation of the appraisal of our methods is that we did not evaluate the views of the stakeholders’ and policy-makers involved in our reviews. Stakeholder satisfaction in our outputs, however, has been evaluated as part of our knowledge mobilisation process and impact assessment, which is reported separately [8].

### Implications for future practice and research

Key learning points are summarised in Box 3. Our rapid review process was developed to support the need for urgent or rapid evidence needs during the COVID-19 pandemic. The same process could support rapid reviews with longer time frames (3–6 months) or more systematic reviews to support policy decision-making. The longer the available timeframe; the more systematic review approaches can be used and less methodological shortcuts are required.

Identifying a specific decision problem is an integral part of the review process. One of the key learning points identified was the need to enhance stakeholders’ abilities to identify focused policy-relevant research questions. The importance of stakeholders in developing and refining the review question, eligibility criteria and outcomes of interest were highlighted by all the key sources included in the best practice framework. Further research is needed to identify the most appropriate methods of engaging stakeholders early in the process to identify evidence needs and how these translate into focussed research questions.

A key limitation in our review process and an important area for further research is identifying, recording, and managing financial conflicts of interest that stakeholders may have. We are not aware of any of our stakeholders having any financial conflicts of interest to date, but we did not routinely collect this information. In going forward we will add an action at the start of each review, for example as part of the first stakeholders meeting, to request that stakeholders disclose any conflict of interest they may have. Our reporting template includes a section on conflicts of interest, but this relates to the authors, and not the stakeholders whose input is generally listed under the acknowledgements. We will look to update our reporting template to comply with the new Reporting Conflicts of Interest and Funding in Health Care Guidelines: The RIGHT-COI&F Checklist, when it is available [40]. An on-going systematic review of existing literature on conflict of interest issues when engaging with stakeholders (including public involvement) in healthcare guideline development, which is part of a wider research project undertaken by the Multistakeholder Engagement (MuSE) working group, will also help address the need for new guidance in this area [41, 42].

Further research is needed to assess the impact of using various methodological short cuts on the validity of rapid review findings. Such research can also provide the basis for minimum standards to minimise inaccuracies and bias, in particular for non-intervention reviews.

The quality (or risk of bias) assessment provides important information on the trustworthiness of the results of included studies. Recent methodological advances in the field of risk of bias assessment (which focuses on internal validity) advocate a move away from the use of critical appraisal tools that cover additional concepts such as imprecision, external validity and reporting [28, 43]. They also recommend that the assessment occurs at domain level, supported by signalling questions, rather than using a checklist approach. An example of which includes the ROBINS-I for non-randomised studies [28]. Existing reviews of quality assessment tools identified numerous tools that can be used in systematic or rapid reviews, but few are designed to cover multiple study designs [44–47] and there is no consensus on the most appropriate tools for rapid reviews [33]. Further work is needed to explore the use of a single tool that covers multiple study designs in rapid reviews of intervention effects [44]. Further work is also needed to develop the optimal approach for selecting appropriate study design descriptors, in the context of a rapid review, of real-world natural experiments or quasi-randomised controlled trials. This is likely to be particularly pertinent when conducting a rapid review of service delivery or public health interventions.

Guidance is required on how to assess the certainty or confidence in the overall body of evidence where the GRADE (or GRADE-CERQual [48]) assessment is difficult. Although it is recommended that assessing the certainty of evidence is based on GRADE for Cochrane rapid reviews of interventions [49], it is also acknowledged that it may not always be easy to implement within either the rapid review [7] or emergency preparedness [50] context.

## Conclusions

Our bespoke review process enabled us to successfully address a high volume of review questions in a timely manner using a transparent and adaptable approach. The collaboration between established research teams in Wales and the strong stakeholder involvement embedded in the review process were considered particular strengths of the overall review process. A number of key learning points were identified, which focussed on: enhancing stakeholders’ abilities to identify focused policy-relevant research questions; the collection and storage of our review protocols at a central location; tightening our quality assurance process regarding study selection, data extraction and risk of bias assessment; the piloting of an algorithm for assigning study design descriptors; and to incorporate, where appropriate, an assessment of the confidence in the overall body of evidence using GRADE or GRADE-CERQual in our reviews.

## Supplementary Information

Additional file 1.

Additional file 2.

## Data Availability

All data relevant to the study are included in the article or uploaded as supplementary materials.

## Abbreviations

COVID-19: Coronavirus disease
GRADE: Grading of Recommendations Assessment, Development and Evaluation
GRADE-CERQual: GRADE-Confidence in the Evidence from Reviews of Qualitative research
PPG: Public Partnership Group
REM: Rapid evidence map
RES: Rapid evidence summary
RR: Rapid review
SAGE: Strategic Advisory Group of Experts on Immunization
TAC: Technical advisory cell
TAG: Technical advisory group
WCEC: Wales COVID-19 Evidence Centre
