# Supplementary material for: A bespoke rapid evidence review process engaging stakeholders for supporting evolving and time-sensitive policy and clinical decision-making: reflection and lessons learned from the Wales COVID-19 Evidence Centre 2021–2023
Source: Health Res Policy Syst. 2025 Mar 20;23:36. doi: 10.1186/s12961-025-01297-w (PMC11927267; doi:10.1186/s12961-025-01297-w)
Supplement: Supplementary file 1 — Additional file 1. [file 12961_2025_1297_MOESM1_ESM.docx]

**A bespoke rapid evidence review process engaging stakeholders for supporting evolving and time-sensitive policy and clinical decision-making: reflection and lessons learned from the Wales Covid-19 Evidence Centre 2021-23**

**RESOURCE LIST**

A list of key resources was developed to guide and track the searches conducted as part of the Rapid Evidence Summary and subsequent Rapid evidence map or Rapid Review. This was developed in collaboration with information scientists (JW, EH) and researchers from the Collaborating Partner groups (HM, SE) and was also informed by the experience gained in producing and maintaining the COVID-19 Evidence digest by HTW (<https://www.healthtechnology.wales/covid-19/covid-19-evidence-digest/>), which was developed by Jenni Washington.

The resources list includes both COVID-19 specific resources and generic resources of robust tertiary or secondary research. The list is not intended to be exhaustive, however, it does include four prioritised resources, which are intended to be searched for all COVID-19 related review questions: Cochrane COVID Review Bank, WHO Global Coronavirus Database, L*OVE COVID, VA-ESP. The list is continually updated to reflect the changing evidence needs relating to the pandemic, with modifications being informed by the experience gained from maintaining the HTW digest and ongoing feedback from the review teams.

**Table A1: Resource list**

| **Resource** | **Success or relevancy of the retrieval** |
| --- | --- |
| **Priority COVID resources for reviews** | |
| [Cochrane COVID Review Bank](https://covidreviews.cochrane.org/search/site)  (Browse list of titles)  https://covidreviews.cochrane.org/search/site | Choose an item. |
| [WHO Global Coronavirus Database - secondary evidence](https://search.bvsalud.org/global-literature-on-novel-coronavirus-2019-ncov/)  (Use filter options to limit search results to secondary evidence under "Type of Study" and English language under "Language")  https://search.bvsalud.org/global-literature-on-novel-coronavirus-2019-ncov/ | Choose an item. |
| [L*OVE COVID - systematic reviews](https://app.iloveevidence.com/loves/5e6fdb9669c00e4ac072701d?population=5e7fce7e3d05156b5f5e032a&classification=systematic-review)  (Links to the systematic reviews section)  https://app.iloveevidence.com/loves/5e6fdb9669c00e4ac072701d?population=5e7fce7e3d05156b5f5e032a&classification=systematic-review | Choose an item. |
| [VA-ESP](https://www.covid19reviews.org/index.cfm)  (Use "search this page" to limit to a concept. A second (or subsequent) concept can be applied to the results list by using "search this page" again.)  https://www.covid19reviews.org/index.cfm | Choose an item. |
| **Additional COVID resources for reviews**  *(Tailor the list according to the topic and potential evidence base. In some cases, it may be preferable to scan the main (generic) source rather than COVID-19 specific product; listed under secondary research)* | |
| [EPPI-Centre](https://eppi.ioe.ac.uk/cms/Projects/DepartmentofHealthandSocialCare/Publishedreviews/COVID-19Livingsystematicmapoftheevidence/tabid/3765/Default.aspx) - Living map of the evidence of studies on COVID-19 identified in MEDLINE and EMBASE, that groups the evidence into broad themes  (Select "Access current version" below first picture)  https://eppi.ioe.ac.uk/cms/Projects/DepartmentofHealthandSocialCare/Publishedreviews/COVID-19Livingsystematicmapoftheevidence/tabid/3765/Default.aspx | Choose an item. |
| [EUnetHTA – COVID 19 response](https://eunethta.eu/services/covid-19/)  (Not a searchable database but lists of evidence reviews covering diagnostics and treatments)  https://eunethta.eu/services/covid-19/ | Choose an item. |
| [Trip](https://labs2020.tripdatabase.com/) – for guidelines  (TripPro can be accessed by an institutional based subscription based via institution, otherwise use Trip)  As a COVID-19 resource for guidelines – search for (covid-19 OR covid19 OR sars-cov-2 OR sars-cov2 OR sarscov2) and the topic/concept of interest, then filter by UK guidelines, covers NICE and SIGN. Can also filter for non-UK guidance.  <https://www.tripdatabase.com/> | Choose an item. |
| ***For topic specific / focused review questions*** | |
| [COVID-END](https://www.mcmasterforum.org/networks/covid-end) – Evidence summaries (McMaster Health Forum)  (Incorporates multiple COVID-19 resources, including many listed here. May be useful for topic specific/focused questions; may not be useful for border questions)  https://www.mcmasterforum.org/networks/covid-end | Choose an item. |
| [COVID-19 Evidence Alerts from McMaster PLUS](https://plus.mcmaster.ca/COVID-19/)^TM^  Usefulness dependent on topic; may not be user friendly for broad/complicated questions  https://plus.mcmaster.ca/COVID-19/ | Choose an item. |
| **Additional COVID resources for primary studies** | |
| [WHO Global Coronavirus Database - primary studies](https://search.bvsalud.org/global-literature-on-novel-coronavirus-2019-ncov/)  (Use filter options to limit search results to primary evidence under "Type of Study" and English language under "Language")  https://search.bvsalud.org/global-literature-on-novel-coronavirus-2019-ncov/ | Choose an item. |
| [L*OVE COVID - primary studies](https://app.iloveevidence.com/loves/5e6fdb9669c00e4ac072701d?population=5e7fce7e3d05156b5f5e032a&classification=primary-study)  https://app.iloveevidence.com/loves/5e6fdb9669c00e4ac072701d?population=5e7fce7e3d05156b5f5e032a&classification=primary-study | Choose an item. |
| [Cochrane COVID-19 Study Register](https://covid-19.cochrane.org/)  https://covid-19.cochrane.org/ | Choose an item. |
| **Secondary resources for reviews relevant to local/UK context** | |
| [United Kingdom Health Security Agency (UKHSA)](https://ukhsalibrary.koha-ptfs.co.uk/covid19rapidreviews/) – COVID-19 Rapid Reviews  https://ukhsalibrary.koha-ptfs.co.uk/covid19rapidreviews/ | Choose an item. |
| [NICE resources for COVID reviews](https://www.nice.org.uk/guidance/conditions-and-diseases/respiratory-conditions/covid19/products?Status=Published)  https://www.nice.org.uk/guidance/conditions-and-diseases/respiratory-conditions/covid19/products?Status=Published  *Any queries regarding ongoing or planned reviews contact Chris Connell:* [*Chris.Connell@nice.org.uk*](mailto:Chris.Connell@nice.org.uk) | Choose an item. |
| [Healthcare Improvement Scotland – COVID-19: Evidence for Scotland](http://www.healthcareimprovementscotland.org/our_work/coronavirus_covid-19/evidence_for_scotland.aspx)  (not a searchable database but a lists Once for Scotland guidance, rapid evidence reviews, NICE rapid guidelines evidence covering diagnostics and treatments)  http://www.healthcareimprovementscotland.org/our_work/coronavirus_covid-19/evidence_for_scotland.aspx | Choose an item. |
| [Ireland, HSE Library, COVID-19 Summaries of Evidence](https://hselibrary.ie/covid19-evidence-summaries/)  not a searchable database but a list of all summaries of evidence that HIQA have been asked to address)  https://hselibrary.ie/covid19-evidence-summaries/ | Choose an item. |
| [HIQA Health Information and Quality Authority (Ireland)](https://www.hiqa.ie/reports-and-publications/health-technology-assessments?tid_1=All&field_hta_topics_target_id=112) – Rapid reviews  https://www.hiqa.ie/reports-and-publications/health-technology-assessments?tid_1=All&field_hta_topics_target_id=112 | Choose an item. |
| [SAGE](https://www.gov.uk/government/organisations/scientific-advisory-group-for-emergencies)  (if relevant)  <https://www.gov.uk/government/organisations/scientific-advisory-group-for-emergencies> | Choose an item. |
| **Secondary resources for reviews produced by key international organisations** | |
| [NCCMT COVID-19 rapid reviews (Canada)](https://www.nccmt.ca/covid-19/covid-19-rapid-evidence-service)  https://www.nccmt.ca/covid-19/covid-19-rapid-evidence-service | Choose an item. |
| [ECDC European Centre for Disease Prevention and Control](https://www.ecdc.europa.eu/en/publications-data) (COVID-19 outputs)  https://www.ecdc.europa.eu/en/publications-data | Choose an item. |
| [CDC Centre for Disease Control and Prevention - Guidance for COVID-19](https://www.cdc.gov/coronavirus/2019-ncov/communication/guidance.html) (US) https://www.cdc.gov/coronavirus/2019-ncov/communication/guidance.html | Choose an item. |
| [AHRQ Agency for Healthcare Research and Quality (US)](https://www.ahrq.gov/coronavirus/health-systems-research.html)  (Note: only 1 of these covid-19 reviews are actively being kept updated as a living review: “Antibody Response Following SARS-CoV-2 Infection and Implications for Immunity: A Living Rapid Review”  https://www.ahrq.gov/coronavirus/health-systems-research.html | Choose an item. |
| [NASEM The National Academy of Sciences Engineering Medicine - Coronavirus Resources Collection](https://www.nap.edu/collection/94/coronavirus-resources) (US)  https://www.nap.edu/collection/94/coronavirus-resources | Choose an item. |
| [Australian National COVID-19 Clinical Evidence Task Force](https://covid19evidence.net.au/) - Living Guidelines; mainly treatment  https://covid19evidence.net.au/  (*also incorporated in Trip*) | Choose an item. |
| **Secondary research resources for (non-COVID-19) reviews**  (*Tailor the list according to the topic and potential evidence base, talk to stakeholder before proceeding with this type of search*) | |
| [Trip](https://labs2020.tripdatabase.com/)  (TripPro can be accessed by an institutional based subscription based via institution, otherwise use Trip)  <https://www.tripdatabase.com/> | Choose an item. |
| [Cochrane Database of Systematic Reviews (CDSR)](https://www.cochranelibrary.com/cdsr/reviews)  https://www.cochranelibrary.com/cdsr/reviews | Choose an item. |
| [Campbell Collaboration](https://www.campbellcollaboration.org/better-evidence.html)  https://www.campbellcollaboration.org/better-evidence.html | Choose an item. |
| JBI (via OVID)  (Subscription based service – WCEBC has a subscription) | Choose an item. |
| [Epistemonikos](https://www.epistemonikos.org/en/advanced_search)  https://www.epistemonikos.org/en/advanced_search  https://www.epistemonikos.org/ (for the simple search) | Choose an item. |
| [International HTA database (INAHTA-HTA)](https://database.inahta.org/)  (for technology & intervention questions only)  https://database.inahta.org/ | Choose an item. |
| [PROSPERO](https://www.crd.york.ac.uk/prospero/)  https://www.crd.york.ac.uk/prospero/ | Choose an item. |
| [PubMed](https://pubmed.ncbi.nlm.nih.gov/)  Filter by systematic reviews, reviews or meta-analysis once search undertaken  https://pubmed.ncbi.nlm.nih.gov/ | Choose an item. |
| **Additional resources searched**  (*Add in any additional resources that have been used, e.g. Scopus, HMIC, Social Care Online)* | |
| [Google Advanced Search](https://www.google.co.uk/advanced_search)  https://www.google.co.uk/advanced_search | Choose an item. |
| [Google Scholar](https://scholar.google.com/)  https://scholar.google.com/ | Choose an item. |
