# Supplementary material for: A bespoke rapid evidence review process engaging stakeholders for supporting evolving and time-sensitive policy and clinical decision-making: reflection and lessons learned from the Wales COVID-19 Evidence Centre 2021–2023
Source: Health Res Policy Syst. 2025 Mar 20;23:36. doi: 10.1186/s12961-025-01297-w (PMC11927267; doi:10.1186/s12961-025-01297-w)
Supplement: Supplementary file 2 — Additional file 2. [file 12961_2025_1297_MOESM2_ESM.docx]

**A bespoke rapid evidence review process engaging stakeholders for supporting evolving and time-sensitive policy and clinical decision-making: reflection and lessons learned from the Wales Covid-19 Evidence Centre 2021-23**

**METHODS USED IN WCEC RAPID REVIEWS AND RAPID EVIDENCE MAPS**

**Table: Summary of methods used in WCEC Rapid Reviews and Rapid Evidence Maps ordered by Collaborating Partner group**

| **Product/ #no** | **Partner group** | **Review*** | **Search date** | **Reviewing approach, *RQ framework used*** scope/limit** | **Searches***** | **Search - hits** | **Study selection** | **Data extraction** | **Quality appraisal (QA)** | **QA tools****** |
| --- | --- | --- | --- | --- | --- | --- | --- | --- | --- | --- |
| **RR_02** | SURE | MH of keyworkers | May 2021 | (Prior *RES included 53 SRs*)  PICO Framework;  UK studies | (*RES: priority C-19 resources*),  Lit covid | 184 records screened plus 103 from SRs/other sources;  30 FT articles;  **20 included studies** | T&A/FT – single reviewer, uncertainties in FT checked | Single reviewer | Single reviewer, uncertainties discussed | JBI design specific critical appraisal tools |
| **RR_13** | SURE | CYP aged 3-13 years | Aug 2021 | (prior *RES: search of C-19 resources id 232 reviews, 28 FT SRs screened, 10 included, but only 1 provided useful findings*)  PICO Framework;  UKs studies (plus 1 RR from RES) | Medline, PsycINFO, Scopus | 536 records plus 38 from other sources);  204 FT articles  **14** **included studies** (1=RR) | T&A/FT – single reviewer, uncertainties in FT checked | Single reviewer | Single reviewer, uncertainties discussed | JBI design specific critical appraisal tools;  Before and after studies: NHLBI, 2013;  RR: Hunter, 2020 |
| **REM_25** | SURE | Disabled people inequalities | Dec 2021 | Scoping review  PCC Framework;  UK, all EHRC domains, 2020 onwards, published/preprints | Medline, PsycInfo, Collabovid,  Organisational websites,  CGs – NICE, SIGN, SC online, Trip | 494 records screened (d/b, guidelines and other sources);  135 FT article;  **68 included studies** | T&A/FT – single reviewer, uncertainties checked | Single reviewer | NA (REM) | NA |
| **RR_25** | SURE | Disabled people inequalities | NA | PCC Framework;  Studies reporting on the EHRC life domain: Health, UK, peer-reviewed studies plus grey -lit reporting new outcomes | (Using prior REM searches) | *68 studies screened (REM includes);*  **19 included studies** (1 was grey literature) | T&A/FT – single reviewer, uncertainties in FT checked | Single reviewer | Single reviewer | JBI-critical appraisal tool for prevalence studies;  MMAT;  CASP for qualitative studies |
| **REM_29** | SURE | LGBTQ+ inequalities | Feb 2022 | Scoping review plus additional evaluation of reported recommendations and actions (summary and critical appraisal of studies presenting implications/ recommendations or evaluated interventions)  PCC Framework; SRs (2016-2022) and UK primary studies (2019-2022) | Priority C-19 resources, Medline, PsycInfo, Embase, Science Citation Index, Relevant websites. | 252 records screened;  75 FT articles;  **35 included** (34 studies, 1 SR) (*13 in the additional evaluation*) | T&A/FT – single reviewer, uncertainties in FT checked | Single reviewer | Single reviewer | JBI design specific critical appraisal tools;  MMAT;  CASP for qualitative studies |
| **RR_37** | SURE | Challenging behaviours amongst learners | July 2022 | PICO Framework;  UK and North Atlantic countries, 2015 onwards | Medline, Scopus, Proquest Education Collection, EBSCOHost BE, grey literature sources, and text mining + citation tracking of Connected Papers, Dimensions, Scopus, Web of Science. | 2312 records screened;  119 FT articles;  ***46 studies*** - all outcomes; **14 studies** of prioritised outcomes. | Single reviewer, where a reviewer was uncertain – checked by second | Single reviewer | Single reviewer | JBI design specific critical appraisal tools; **GRADE** (for appraising body of evidence) |
| **RR_39** | SURE | Workforce capacity for hospital discharge of older adults | May 2022 | PCC Framework;  UK, 2020 onwards, published/preprints | Medline, HMIC, SCOPUS and Social Policy & Practice, Epistemonikos, Google Scholar, 5 grey literature sources | 198 records screened;  87 FT articles;  **19 studies** (11 SRs and 8 primary studies) | T&A/FT – single reviewer, uncertainties checked by second | Single reviewer | Single reviewer | AMSTAR 2;  CASP for qualitative studies;  JBI design specific critical appraisal tools.  **GRADE** assessment within SRs reported |
| **RR_03** | BIHMR | GI cancer backlog | June 2021 | PICO Framework;  Published UK studies | Medline, Embase, ASSIA, CINAHL Cochrane Library, HTA libraries, MEDLINE, Prospero, | 395 records screened;  15 FT articles;  **9 RRs included** | T&A/FT – two reviewers; disagreements discussed | Single reviewer with 1/4 checked by a second reviewer | Single reviewer with 1/4 checked by a second reviewer | JBI design specific critical appraisal tools |
| **RR_18** | BIHMR | IPAC in care homes and domiciliary care | Sept 2021 | PICO Framework;  OECD countries, 2020-21 | Medline, ASSIA, CINAHL, Cochrane Library, HTA libraries, Prospero | 1868 records screened;  17 FT articles;  **15 includes** (14 studies and 1 RR) | Not stated | Single reviewer, sample checked by a second. A proportion was double extracted to check for discrepancies | Not stated | MMAT;  CEBM checklist for surveys;  JBI design specific critical appraisal tools |
| **RR_27** | BIHMR | Gender inequalities | Dec 2021 | PICO Framework;  OECD countries, 2020-21 | Medline, CINAHL, PsycInfo, Cochrane Library, ASSIA, EMBASE | 1291 records screened;  100 FT articles;  **21 includes** (8 studies, 6 commentaries, and 7 reviews) | Single reviewer, sample screened by second reviewer | Single reviewer with 10% double extracted to check for discrepancies | Single reviewer with 1/4 checked by second reviewer, discrepancies discussed by team | JBI design specific critical appraisal tools |
| **RR_40** | BIHMR | Long COVID morbidity | June 2022 | Two published SRs used as the starting point; primary studies in the SRs were screed for inclusion it the RR.  PICO Framework;  2021-22 | ASSIA, CINAHL, Cochrane Library, EMBASE, Medline, PsycInfo | 1008 records screened;  48 FT articles;  **15 included** | T&A/FT – three reviewers  FT - not stated  Queries discussed between the team | Single reviewer with 10% double extracted to check for discrepancies | Single reviewer with 1/4 checked by second reviewer, discrepancies discussed by the team | JBI design specific critical appraisal tools |
| **RR_42** | BIHMR | **Interventions to support long COVID** | Sept 2022 | Review of CGs and SRs identified in preliminary scoping search (part of RES) plus review of primary studies focusing on interventions to support return to normal activities of living  PICO Framework;  OECD countries, 2012-22 | Medline, ASSIA, PsycINFO, CINAHL, Embase, Cochrane Library | 664 records screened;  21 FT articles;  **4** included **primary studies, 3 GCGs, 3 SRs** | T&A/FT – screening conducted by two reviewers, with sample double screened  FT - not stated  Queries discussed between the team | Single reviewer with 10% double extracted to check for discrepancies | Single reviewer with 1/4 checked by second reviewer, discrepancies discussed by the team | JBI design specific critical appraisal tools |
| **RR_07** | HTW | Face coverings (update) | July 2021 | (Prior *RES – search of* *C-19 priority resources and iHTA d/b id 18 reviews)*  Update of the 2 most complete and up to date RRs (*PHE 2021: n=31 studies; Chou, 2020: n=39*).***  PICO Framework (aligned with PHE definitions);  Studies published after Sep 2020 (date of most recent PHE RR search) | Medline, Emabse, Cochrane library, Cochrane C-19 study register | 2336 records screened;  53 FT articles (plus 4 reviews from RES);  **4 includes** (2 RRs and 2 new primary studies | Single reviewer | Single reviewer with sample checked by a second reviewer | No formal QA; utilised QA used by RRs. Single reviewer provided narrative summary of key limitations of included primary studies | NA |
| **RR_36** | HTW | NPI Tool | Dec 2021 | Aim – select robust modelling studies that can be used with NPI-tool  Summary and critique of prioritised modelling studies published since 2021  PICO Framework | Medline, Emabse, Cochrane C-19 study register, L*OVE COVID, PROSPERO,  (*no date limit on searches*) | 866 records screened;  44 FT articles;  **9 includes** | T&A Single reviewer. FT single reviewer with selection decisions checked by a second | Single reviewer | Initial pilot (n=3) conducted by two independent reviewers. Remainder completed by one reviewer and checked by a second | Modelling studies: Burns, 2021 |
| **RR_12** | HTW | Transmission in vaccinated populations | Oct 2021 | (*RES id 3 RR, 1 CG, 1 protocol, 1 economic evaluation*)  Update of an existing robust RR (review period up Aug 2021); searched for primary studies published Aug-Oct 2021  PICO Framework | Medline, Emabse, Cochrane library, Cochrane C-19 study register, INAHTA, Clinical trials, WHO trial register, PROSPERO  NICE, Trip, Google | 7,823 records screened;  72 FT articles;  36 includes  (1 RR; **35 primary studies**, 9 were new/not in RR) | Three reviewers checked eligibility | Single reviewer with sample checked by a second | Observational studies - independent assessment by two reviewers, differences discussed, and joint agreement reached.  RCTs and RR – single reviewer | RCTs: ROB2;  Observational studies: ROBINS-I;  RR: ROBIS |
| **RR_20** | HTW | Financial support for isolation | April-May 2022 | Preliminary RES identified 2 RRs and 1 SR)  Existing reviews used to identify relevant primary studies and searches focused on primary studies published after the review searches  PICO Framework; 2021- | Medline, Embase, Priority C-19 resources, Cochrane C-19 Study Register, Cochrane Library, Epistemonikos, Scopus, WHO ICTRP, clinicaltrials.gov, PROSPERO, ABI Inform | 3,657 records screened;  45 FT articles;  **9 includes** (5 from existing RRs/SR and 4 published after reviews) | Not stated; early primary studies identified via existing RRs/SR | Single reviewer with consistency checks carried out by a second | No formal QA conducted; potential limitations of included studies discussed in narrative synthesis | NA |
| **RR_41** | HTW | Air filtration in hospitals | July 2022 | Prioritised existing reviews and primary studies conducted in real-world healthcare settings, but due to the lack of available data in this context, modelling and experimental (controlled environment) studies that met the eligibility criteria also included.  PICO Framework | Medline, Emabse, WHO Global Coronavirus database, L*VE COVID, Cochrane C-19 study register, VA-ESP, Cochrane library, Scopus, PROSPERO | 6,220 records screened;  144 FT articles;  **7 includes** | T&A Single reviewer with sample checked by a second and disagreements resolved by consensus among 3 reviewers.  FT Single reviewer with sample checked by a second and disagreements resolved by consensus among 3 reviewers | T&A / FT: Single reviewer with sample checked by a second and disagreements resolved by consensus among 3 reviewers. | Not stated | SRs/RRs: ROBIS; Observational studies: NHLBI design specific tool; Modelling studies: Burns, 2021 |
| **RR_46** | **HTW** | **PPE in Community settings** | Nov 2022 | Prioritised SRs / RRs, but none focused on the setting of interest. Searches therefore extended to include primary studies reporting on GP or ambulance settings specifically.  PICO Framework;  2020-22 | Medline, Emabse, Cochrane library, Epistemonikos, Trip Pro, INAHTA HTA, WHO ICTRP, clinicaltrials.gov, PROSPERO | 7,675 records screened;  167 FT articles;  **7 includes** | T&A Single reviewer.  FT Single reviewer with decisions checked by a second and disagreements resolved by consensus. | Single reviewer checked by a second | Not stated | SRs/RRs: ROBIS; Observational studies: NHLBI tool for observational and cross-sectional studies |
| **RR_11** | PHW | IPAC in schools | July 2021 | Summary and appraisal of a living RR by NCCMT (*id during RES*).  PICO Framework;  Reviews, OECD countries | *Updated RES search;*  Priority C-19 resources;  LitCovid (reviews and primary), Trip, CDSR, Campbell, JBI, Epistemonikos, PROSPERO, Medline, HTW C-19, PHE C-19, NICE C-19, Ireland C-19 ES, CADTH, HIQA, CDC, NCCMT, Google Advance, | 54 records screened;  21 FT articles;  **10 includes** (2 RRs and *8 Protocols*);  Focused on **1 LRR** as it superseded others | T&A: one reviewer;  FT: two reviewers | One reviewer performed data extraction and two independent reviewers carried out consistency checking | One reviewer conducted the QA and a second reviewer performed a consistency check | AMSTAR 2 |
| **RR_08** | PHW | Surgical backlog (demand-side) | Sept 2021 | Existing reviews (id during RES) used to id primary studies, supplemented by search for primary studies of specific intervention (COVID-light sites)  PICO Framework;  Primary studies (observational and qualitative), OECD countries | *Searches for reviews conducted during RES:*  Priority C-19 resources;  LitCovid (reviews and primary), EPPI- map, ITS-HTA, EUnetHTA, Trip, CDSR, Campbell, JBI, Epistemonikos, PROSPERO, PubMed, PHE C-19, NICE C-19, HCI Scotland C-19, Ireland C-19 ES, CADTH, HIQA, CDC, AHRQ, NCCMT, WHO C-19, Google Advance, Medline;  *Additional search for COVID-light sites:*  C-19 Research database (via Dialog) and Google advanced | 215 records screened (6 reviews: n= 56 studies; additional searches: n=156);  35 FT articles;  **17 reviews included** | T&A: independently in duplicate, by two reviewers  FT: independently in duplicate, by two reviewers | One reviewer and checked by a second reviewer | One reviewer, with verification of all judgements by a second reviewer | RCTs: ROB2;  Non-randomised Studies of interventions tool: ROBINS-I |
| **RR_30** | PHW | Surgical waiting list | Feb 2022 | RoRs  Large vol of relevant reviews (n=57), first prioritised using essential criteria for SRs (n=42) then grouped by intervention type and prioritised further using robustness/coverage criteria for inclusion in the narrative synthesis. All SRs data extracted and included in a map (matrix) outlining interventions and outcomes assessed.  PICO Framework;  SRs, OECD countries, 2011 - | *Searches for reviews conducted during RES and updated:*  Priority C-19 resources;  LitCovid (reviews and primary), EPPI-map, ITS-HTA, EUnetHTA, COVID-END, C-19 McMaster, LOVE primary, Cochrane C-19 register, UKHSA C-19, NICE C-19, HCI Scotland C-19, Ireland C-19 ES, HIQA, SAGE, NCCMT, ECDC, CDC, AHRQ, NASEM, Australia NC-19CETF, Trip, CDSR, Campbell, Epistemonikos, PROSPERO, WHO C-19, Google Advance, Medline,  *Scan of References,*  *Additional search in MEDLINE for SRs on social prescribing* | 659 records screened;  140 FT articles;  **58 included** (48 reviews, *10 Protocols*)  17/42 SRs prioritised for narrative synthesis | T&A / FT: independently in duplicate, by three reviewers. | Two reviewers | One reviewer, with verification of all judgements by a second | AMSTAR 2 |
| **RR_35** | PHW | Cancer screening | May 2022 | Focused on primary studies conducted during or post C-19 pandemic. Pre-pandemic literature (used for comparison) identified via supplementary scoping search for SRs  PICO Framework; limited to countries with comparable screening programmes, 2020-22 | Priority C-19 resources; Cochrane COVID-19 Study Register; Medline; PsycINFO, Cochrane Library; clinicaltrials.gov; WHO ICTRP; Trip – for guidelines, Google Advanced Search, Google Scholar | 5,165 records screened;  ? FT articles;  **3 includes** | T&A: Single reviewer.  FT: Single reviewer with consistency checks of included studies conducted by a second. Disagreements resolved by 3rd reviewer | Single reviewer with consistency checks conducted by a second | Single reviewer with verification of all judgements by a second. Discrepancies resolved by the team | Qualitative studies: CASP;  SRs: AMSTAR 2 |
| **REM_43** | PHW | Community diagnostic centres | July-Aug 2022 | Review of primary studies  PICO Framework | Priority C-19 resources, UKHSA (C-19), NICE resources (C-19), Healthcare Improvement Scotland (C-19), Ireland, HSE Library (C-19), HIQA, SAGE, NCCMT (C-19), ECDC (C-19), CDC (C-19), AHRQ, NASEM (C-19), Australian National C-19 Clinical Evidence Task Force, MEDLINE; Embase, Trip, Cochrane Library, Campbell, Epistemonikos, PROSPERO, Google Advanced Search, Google Scholar, Google | 3,603 records screened;  ? FT articles;  **50 includes** | T&A: Single reviewer.  FT: Single reviewer with consistency checks of included studies conducted by a second. Disagreements resolved by 3rd reviewer | Single reviewer | NA | NA |
| **RR_43** | PHW | Community diagnostic centres | July-Aug 2022 | Narrower focus than initial REM | Studies identified via REM searches | FT articles (from REM)  **20 included studies** | Two independent reviewers | Single reviewer with consistency checks conducted by a second | Single reviewer with verification of all judgements by a second. Discrepancies resolved by the team | JBI design specific critical appraisal tools |
| **RR_04** | WCEBC | Healthcare education | June 2021 | PICO Framework;  Quantitative studies, OECD countries, 2019-21 | MEDLINE, Embase, CINAHL, ERIC | 7,127 records screened;  157 FT articles;  **23 studies included** | Two reviewers dual screened 20% of the citations; remainder categorised as include (*uncertain classed as include*) or exclude by single reviewer;  FT screened by one reviewer with excludes checked by a second | One reviewer, and checked by another | One reviewer, and judgements verified by a second | JBI design-specific critical appraisal tools;  **GRADE** (for appraising body of evidence) |
| **RR_16** | WCEBC | CYP aged 16-19 years | Aug 2021 | RoRs  Presented as a research map due to volume of research  PICO framework;  No date restriction, any country | SCOPUS, Web of Science, ASSIA, ERIC, BEI,  C-19 specific databases: VA-ESP, L*OVE COVID19, Collabovid and LitCOVID;  18 organisational websites;  Reference lists screened plus forward citation tracking performed using Web of Science | 1,390 records screened;  44 FT reports;  **23 includes**  (14 SRs, *1 protocol,* 3 RRs, 5 organisational reports) | Two reviewers dual screened 20% of the citations; remainder categorised as include (*uncertain classed as include*) or exclude by single reviewer;  FT screened by one reviewer with excludes checked by a second | One reviewer and checked by another | Two reviewers with disagreements resolved by a third person | SR: AMSTAR-2  RR: RaPeer tool |
| **RR_26** | WCEBC | SC recruitment/ retention | Nov 2021 | UK studies, 2021  SPIDER framework;  Any study design - qualitative, quantitative, or mixed methods; Evaluation of characteristics, views, experiences | Medline, Social Policy and Practice, Scopus | 1,835 records screened; 81 FT reports;  **39 includes**  (25 studies, 11 organisational reports, and 3 reviews) | T&A: dual screened at least 20%; remainder categorised as include or exclude by single reviewer with at least 50% of excludes screened by a second reviewer.  FT: two independent reviewers | One reviewer and checked by second | One reviewer, judgements verified by second reviewer | MMAT |
| **REM_28** | WCEBC | NHS workers recruitment/ retention | Jan 2022 | Initial REM based on abstracts  PICoS framework;  OECD countries; last 10 years; any study design included, but robust reviews prioritised  Evidence categorised by the phenomena of interest and different clinical staff groups (types) | Priority C-19 resources, LitCovid, EPPI-map, UKHSA C-19, NICE C-19, HCI Scotland C-19, Ireland C-19 ES, HIQA, SAGE, NCCMT, AHRQ, NASEM, Trip, CDSR, JBI, Epistemonikos, PROSPERO, PubMed,  Medline, Cinahl, Google Advance,  websites of 50 key third sector and government organisations  References of RoRs and RRs scanned | **81 includes**  Included: 35 SRs/RSs, 11 narrative reviews, 7 scoping reviews, 5 RoRs, 18 primary studies, and 5 organisational reports or websites | T&A: screened by single reviewer;  FT: screened by two reviewers with disagreements resolved by a third reviewer | For evidence on return to practice: one reviewer and checked for accuracy by a second;  For remaining sections: one reviewer - from abstracts; where information was missing or incomplete full-text consulted | NA (REM) | NA |
| **RR_28** | WCEBC | NHS workers recruitment/ retention | Feb 2022 | RoRs  PICoS framework;  2015 onwards, any country | Medline, Embase, Emcare, HMIC, Cumulative Index of Nursing and Allied Health Literature, Epistemonikos, CENTRAL,  websites of key third sector and government organisations | 1,916 records screened;  41 FT reports;  **9 reviews included** | T&A: dual screened all citations;  FT: two independent reviewers | One reviewer and checked by another | One reviewer and checked by a second | JBI critical appraisal checklist for systematic reviews and research syntheses |
| **RR_32** | WCEBC | Telemedicine | May 2022 | Review of primary studies conducted during or after the C-19 pandemic  PICO framework; high income countries, 2021-2022 | Medline, Embase, CINAHL; WHO Global Coronavirus Database (primary studies), L*OVE COVID (primary studies), Cochrane COVID-19 Study Register, citation tracking via Google Scholar | 5,623 records screened;  62 FT reports;  **14 studies included** | T&A: two reviewers dual screened at least 20% of citations resolving all conflicts when needed  FT: One reviewer and checked by a second | One reviewer and checked by another | one reviewer with judgements verified by a second | JBI design specific critical appraisal tools; **GRADE** (for appraising body of evidence) |
| **REM_45** | WCEBC | Gender inequalities in health and social care | Sept 2022 | Searches targeted specific topic areas of priority  PiCoS framework; high income countries, 2012-, 2018-, or 2021 (depending on topic) -2022 | Medline, Embase, APA PSYCinfo, CINAHL | - | T&A: One reviewer  FT: One reviewer | Conducted by one reviewer | NA (REM) | NA |

**Abbreviations:**

AHRQ Agency for Health care Research and Quality; AMSTAR Assessing the Methodological Quality of Systematic Reviews; CA critical appraisal; CADTH Canadian Agency for Drugs and Technologies in Health; CASP Critical Appraisal Skills Programme; CENTRAL Cochrane Central Register of Controlled Trials; CDC Centers for Disease Control and Prevention; CDSR Cochrane Database of Systematic Reviews; CEBM Center for Evidence Based Management; C-19 COVID-19; CYP children and young people; CG clinical guideline; d/b database; EHRC Equality and Human Rights Commission; ES Evidence synthesis; FT full text; GI gastrointestinal; GRADE Grading of Recommendations Assessment, Development and Evaluation; HCI Healthcare Improvement; HIQA Health Information and Quality Authority; HMIC Health Management Information Consortium; HTA Health Technology Assessment; INAHTA International Network of Agencies for Health Technology Assessment; iHTA international Health Technology Assessment; IPAC Infection Prevention and Control; id identified; JBI Joanna Briggs Institute; LGBTQ+ lesbian, gay, bisexual, transgender, and queer (or questioning); MH mental health; MMAT Mixed Methods Appraisal Tool; NA not applicable; NICE National Institute of Clinical Excellence; NCCMT National Collaborating Centre for Methods and Tools; NHLBI National Heart, Lung and Blood Institute; NPI Non-pharmaceutical interventions; OECD Organisation for Economic Co operation and Development; PCC Population (or Participants), Concept, and Context; PHE Public health England (now known as UK Health Security Agency, UKHSA); PICO Population, Intervention (exposure), Outcome; PICoS Population, Phenomena of Interest, Context and Study design; QA quality assessment; RaPeer Rapid Peer Reviewer Checklist for Rapid Reviews; REM Rapid Evidence map; RES Rapid Evidence Summary; RCT randomised controlled trial; RQ review question; RR rapid review; ROB Risk of bias ; ROBINS-I risk of bias in non-randomized studies of interventions; ROBIS risk of bias in systematic reviews; RoRs Review of reviews (or Umbrella reviews); ScR scoping Review; SPIDER Sample, Phenomenon of Interest, Design, Evaluation, Research type; SR systematic review; T&A titles and abstract; UK United Kingdom; VA-ESP Veterans Affairs Evidence Synthesis Program; WHO World Health Organization; WHO ICTRP World Health Organization International Clinical Trials Registry Platform.

**Footnotes:**

***Two WCEC Rapid Reviews are not included here:**

One review (RR_30), on the impact of COVID-19 induced changes on greenhouse gas emissions, was conducted by the Bangor BioComposites Team (not one of the WCEC Collaborating partners); and the second (RR_44), on strategies to support young people aged 16-19 years, was an update of a previous review (RR_16) using the same methods.

****Frameworks used to format the review question and inform eligibility criteria**

PICO: Population, Intervention (exposure), Outcome

PICoS: Population, Phenomena of Interest, Context and Study design

PCC (*for JBI scoping reviews*): Population (or Participants), Concept, and Context.

SPIDER: Sample, Phenomenon of Interest, Design, Evaluation, Research type

*****Priority COVID-19 resources** include the following, which are generally searched as part of the preliminary Rapid Evidence Summary phase: Cochrane COVID Review Bank, WHO Global Coronavirus Database - secondary evidence, L*OVE COVID - systematic reviews, VA-ESP (see Appendix 1).

*****References**:

Burns J, Movsisyan A, Stratil JM, et al. (2021) International travel‐related control measures to contain the COVID‐19 pandemic: a rapid review. Cochrane Database of Systematic Reviews. 3(CD013717). doi: 10.1002/14651858.CD013717.pub2

Chou R, Dana T, Jungbauer R, et al. (2020c) Masks for prevention of respiratory virus infections, including SARS-CoV-2, in health care and community settings : a living rapid review. Annals of Internal Medicine. 173(7): 542-55. doi: <http://dx.doi.org/10.7326/M20-3213>

Hunter J. (2020). Rapid Peer Reviewer Checklist for Rapid Reviews – RAPeer. Advances in Integrative Medicine. 7(4): 183-6. doi: https://doi.org/10.1016/j.aimed.2020.07.003

National Heart, Lung and Blood Institute (NHLBI) (2013). Quality Assessment Tool for Before-After (Pre-Post) Studies With No Control Group Available from: <https://www.nhlbi.nih.gov/health->topics/study-quality-assessment-tools [Accessed: 24 September 2021]

Public Health England (PHE). (2021) Face coverings in the community and COVID-19: a rapid review (update 1). PHE COVID-19 Rapid Reviews. Public Health England. Available at: https://phelibrary.koha-ptfs.co.uk/covid19rapidreviews/ [Accessed 24 May 2023].

Shea BJ, Reeves BC, Wells G, et al. (2017) AMSTAR 2: a critical appraisal tool for systematic reviews that include randomised or non-randomised studies of healthcare interventions, or both. BMJ, 358:j4008. https://www.bmj.com/content/358/bmj.j4008:

Sterne JA, Hernán MA, Reeves BC, et al. (2016) ROBINS-I: a tool for assessing risk of bias in non-randomised studies of interventions. BMJ, 355. doi: 10.1136/bmj.i4919

Sterne JA, Savović J, Page MJ, et al. (2019) RoB 2: a revised tool for assessing risk of bias in randomised trials. BMJ, 366. doi: 10.1136/bmj.l4898

Aromataris E, Munn Z (Editors). JBI Manual for Evidence Synthesis. JBI (2020). Available from https://synthesismanual.jbi.global. https://doi.org/10.46658/JBIMES-20-01

JBI Critical appraisal tools Avaialble from: https://jbi.global/critical-appraisal-tools [Acessed 24 May 2023]

Hong Q, Pluye P, Fàbregues S, et al. (2018) Mixed Methods Appraisal Tool (MMAT), version 2018. Registration of Copyright (#1148552). Canadian Intellectual Property Office. Available at:

<http://mixedmethodsappraisaltoolpublic.pbworks.com/w/file/fetch/127916259/MMAT_2018_criteria-manual_2018-08-> [Acessed 24 May 2023]
